# A US-Based National Virtual Cohort of People Living With HIV at Risk for Viral Nonsuppression: Protocol for the EPI-LoVE Prospective Cohort Study

**DOI:** 10.2196/108764

**Published:** 2026-10-07

**Authors:** Marjan Javanbakht, Lisa B Hightow-Weidman, Sean D Young, Nora E Rosenberg, Kathryn E Muessig, Sarah Schoetz Dean, Aimee Rochelle, Warren Scott Comulada, Pamina M Gorbach

**Affiliations:** 1 Department of Epidemiology, Fielding School of Public Health, University of California Los Angeles, 650 Charles Young Drive, CHS 21-293, Los Angeles, CA, 90095, United States, 1 310-825-3234; 2 Institute on Digital Health and Innovation, College of Nursing, Florida State University, Tallahassee, FL, United States; 3 Department of Emergency Medicine, UCI School of Medicine, University of California, Irvine, Irvine, CA, United States; 4 Department of Health Behavior, Gillings School of Global Public Health, University of North Carolina at Chapel Hill, Chapel Hill, NC, United States; 5 Department of Psychiatry, UCLA David Geffen School of Medicine, , CA, United States

**Keywords:** HIV, viral suppression, digital cohort, virtual cohort, mobile health, mHealth

## Abstract

**Background:**

Despite advances in HIV treatment, many people living with HIV fail to achieve or maintain viral suppression. Individuals who experience interruptions in HIV care, adherence challenges, and viral nonsuppression are often underrepresented in clinic-based cohort studies. Virtual cohort methodologies offer opportunities to recruit, engage, and retain these populations while integrating longitudinal behavioral, biomarker, and digital engagement data needed to characterize dynamic changes in HIV care engagement and viral suppression.

**Objective:**

The Exploring, Predicting, and Intervening on Long-Term Viral Suppression Electronically (EPI-LoVE) study was developed to establish a national virtual cohort of people living with HIV with current viral nonsuppression or at an elevated risk of future viral nonsuppression. We describe the study design, recruitment, data collection methods, retention strategies, and baseline cohort characteristics.

**Methods:**

Participants were recruited through digital advertising, a national HIV care provider network, and referrals from previous research studies. Eligible participants were adults living with HIV who were currently virally unsuppressed or considered at elevated risk of future viral nonsuppression based on a history of detectable HIV viral load, recent missed HIV care appointments, or a qualifying mental health diagnosis. Following online screening, participant eligibility was confirmed through telephone verification and supporting documentation. Participants completed online surveys every 3 months and HIV viral load assessments every 6 months over 24 months. Data were collected through a study-specific mobile health platform integrating longitudinal behavioral surveys, remote biomarker collection, app engagement metrics, and frequent “Speak Up” microassessments of time-varying behavioral and psychosocial factors associated with HIV care engagement and viral suppression.

**Results:**

The study was funded in May 2023. Participant recruitment occurred between February 2024 and June 2025, and longitudinal data collection is ongoing, with study findings anticipated in spring 2028. A total of 4789 individuals expressed interest in the study, 2739 completed screening, and 2262 met eligibility criteria. A total of 1109 consented, and 1051 enrolled. Among the 2262 eligible participants, 63.4% (n=1435) qualified based on a detectable viral load during the previous 12 months, whereas the remainder qualified based on recent missed HIV care appointments or a qualifying mental health diagnosis. Among the 1051 participants, the cohort was predominantly Black or African American (n=677, 64.4%), with a median age of 36 (IQR 31‐43) years; 70.8% (n=744) reported recent substance use, and 63.7% (n=669) screened positive for moderate-to-severe depressive symptoms. Baseline HIV viral load data were obtained from 863 (82.1%) participants.

**Conclusions:**

EPI-LoVE demonstrates the feasibility of recruiting, verifying, and engaging a geographically diverse national virtual cohort of people living with HIV at elevated risk of viral nonsuppression. By integrating longitudinal behavioral assessments, biomarker collection, and digital engagement data within a single platform, EPI-LoVE provides a scalable framework for studying the dynamic determinants of HIV care engagement and informing future personalized digital interventions.

## Introduction

Achieving and maintaining viral suppression remains a central goal of HIV treatment and prevention efforts. Sustained viral suppression improves individual health outcomes, reduces HIV-related morbidity and mortality, and effectively eliminates the risk of HIV transmission [1,2]. Although effective antiretroviral therapy is widely available in the United States, substantial gaps in viral suppression persist, particularly among racial and ethnic minorities, people who inject drugs, adolescents and young adults, and individuals residing in rural communities [3]. These inequities are driven by a complex interplay of individual, social, structural, and health care–related factors, including poverty, housing instability, stigma, violence, substance use, mental health conditions, and inconsistent access to HIV care [4-9].

Longitudinal studies have shown that viral suppression is not a static outcome and that, while many people living with HIV achieve viral suppression at some point, substantially fewer maintain durable suppression over time [9]. These transitions are influenced by multiple time-varying factors, including substance use, mental health conditions, housing instability, and changes in social support [10-12]. Moreover, these factors frequently co-occur and interact, producing a greater impact on HIV outcomes than any single risk factor alone [13-16]. Understanding these dynamic transitions requires longitudinal approaches capable of capturing changes in behavioral, psychosocial, clinical, and structural determinants of HIV care over time.

Traditional clinic-based cohort studies have generated critical insights into HIV disease progression and treatment outcomes. However, these cohorts often face challenges related to recruitment, geographic reach, participant burden, and long-term retention. Furthermore, because recruitment is clinic-based, these cohorts are also susceptible to selection bias, disproportionately enrolling individuals who are already engaged in care while underrepresenting those at greatest risk for treatment interruptions and viral nonsuppression. Digital cohorts offer opportunities to overcome many of these barriers through remote recruitment, data collection, and participant engagement [17]. By reducing the need for in-person visits, virtual cohorts can facilitate participation among populations that may be difficult to reach, including individuals residing in rural areas, those with transportation limitations, and those who experience stigma or competing life demands that interfere with clinic-based participation. Beyond improving accessibility, virtual cohorts also enable more frequent collection of self-reported information and novel forms of digital data, including measures of participant engagement with study activities and other digital behaviors that may provide insights into changes in HIV care engagement over time.

The Exploring, Predicting, and Intervening on Long-Term Viral Suppression Electronically (EPI-LoVE) study was developed to address several important gaps by establishing a national virtual cohort of people living with HIV who were not currently virally suppressed, had a history of viral nonsuppression, or were at risk of viral nonsuppression. Unlike traditional clinic-based cohorts that rely on less-frequent study visits, EPI-LoVE combined repeated survey assessments, longitudinal HIV viral load measurements, and continuous digital engagement data collected through a study-specific mobile health (mHealth) app. In addition to serving as a longitudinal research cohort, EPI-LoVE provided a platform for developing, evaluating, and ultimately delivering tailored digital interventions to individuals at the greatest risk of poor HIV care outcomes. Guided by the Gelberg-Andersen Behavioral Model for Vulnerable Populations, EPI-LoVE was designed to examine how predisposing, enabling, and need-related factors influence movement through the HIV care continuum and contribute to dynamic changes in viral suppression over time (Figure 1) [18]. The primary objectives of the study are as follows: (1) establish and retain a geographically and demographically diverse national cohort of people living with HIV at elevated risk of viral nonsuppression; and (2) characterize longitudinal patterns of HIV care engagement and viral suppression using integrated behavioral, biomarker, and digital data. This paper describes the study design, recruitment strategy, enrollment procedures, participant verification methods, and baseline characteristics of the EPI-LoVE cohort.

**Figure 1.**
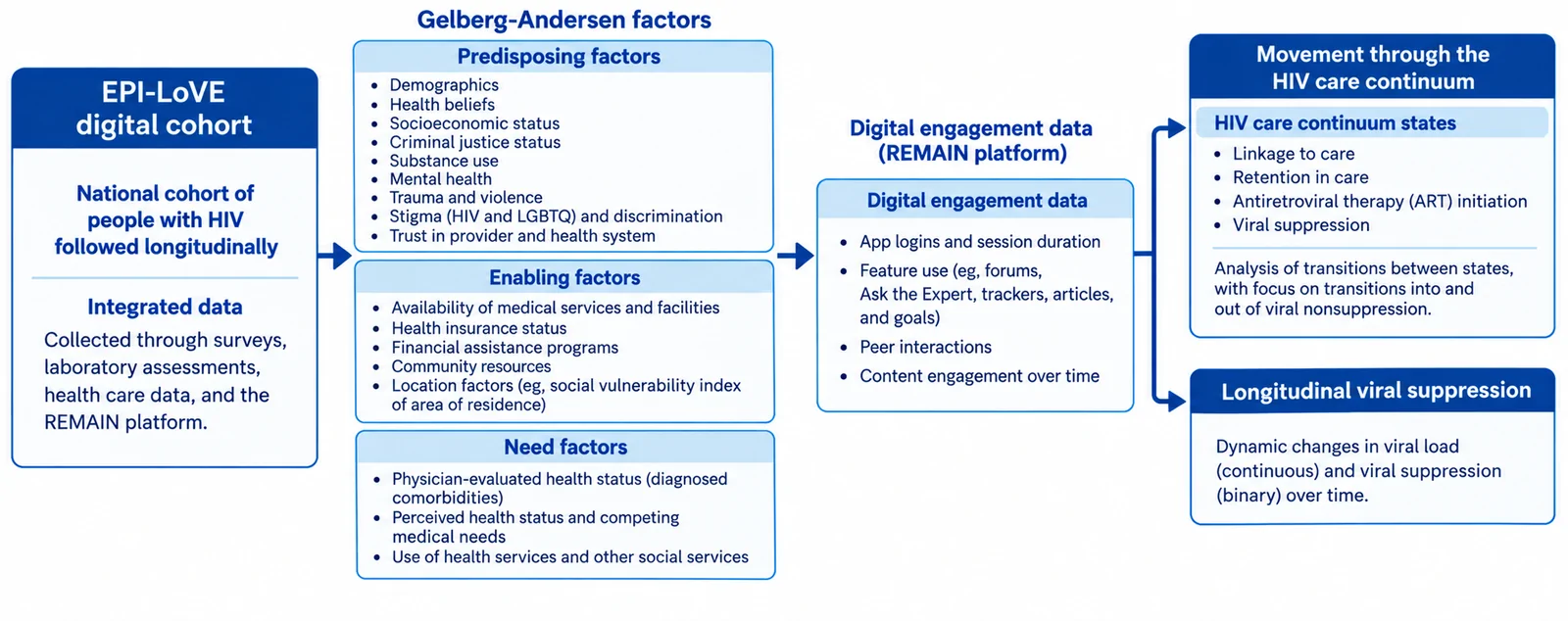
Conceptual framework for the Exploring, Predicting, and Intervening on Long-Term Viral Suppression Electronically (EPI-LoVE) cohort based on the Gelberg-Andersen Behavioral Model for Vulnerable Populations. LGBTQ: lesbian, gay, bisexual, transgender, and queer; REMAIN: REetain, MAintain, and SustaIN.

## Methods

### Study Design

EPI-LoVE is an ongoing, longitudinal, virtual cohort study of 1051 adults living with HIV throughout the United States. Participants are followed for up to 24 months through a combination of online surveys, mobile app engagement, and repeated HIV viral load assessments. The study was designed to prioritize recruitment of populations that experience challenges across the HIV care continuum and are at increased risk of suboptimal HIV care outcomes, including residents of rural communities, younger adults, and people who use substances. Participants were eligible if they met the following criteria: (1) were ≥18 years of age; (2) were living with HIV; (3) were at risk for viral nonsuppression, defined as having at least one of the following: a self-reported detectable HIV viral load during the previous 12 months; suboptimal HIV care engagement, defined as one or more missed HIV care appointments during the previous 12 months; or a diagnosis of generalized anxiety disorder, major depressive disorder, or schizoaffective disorder; (4) had access to a personal smartphone; (5) were willing to participate in longitudinal follow-up procedures; and (6) were willing to provide informed consent. Individuals who were consistently virally suppressed and not considered at an elevated risk for future viral nonsuppression were excluded.

### Recruitment Procedures

Participants were recruited between February 2024 and June 2025 using a multimodal recruitment strategy—including digital recruitment, clinic-based recruitment, and recruitment through existing studies of people living with HIV who expressed interest in other research opportunities—all strategies designed to maximize geographic reach and demographic diversity. The digital recruitment strategy used targeted advertising campaigns across social media platforms and other web-based strategies such as online dating platforms. Recruitment materials were tailored to specific demographic and geographic populations and were optimized using real-time performance analytics. Advertising campaigns were continuously monitored and refined to maximize recruitment efficiency and improve representation among priority populations. Clinic-based recruitment was through a national HIV care provider, which has clinics located throughout the United States. Clinic staff used electronic health record data to identify patients who met study eligibility criteria. Potentially eligible patients were contacted by clinic staff and informed about the study. Those interested were referred to the study website, where they completed the same online screening and eligibility determination process used for all study participants, regardless of recruitment source. Finally, additional recruitment occurred through collaborating research studies conducted at participating institutions. Individuals who had previously indicated willingness to be contacted regarding future research opportunities were invited to complete the EPI-LoVE screening process.

### Participant Verification

Regardless of recruitment source, all individuals expressing interest in the study were directed to the study website, where they could obtain additional information about study procedures and access a secure online portal to begin eligibility screening. Because all study procedures were conducted remotely, a multistage screening and verification process was implemented to protect data quality and prevent duplicate or fraudulent enrollment. The initial online prescreener incorporated CAPTCHA verification, bot detection algorithms, IP address monitoring, and consistency checks across demographic identifiers (eg, initials, date of birth, age, and geographic location). Responses exhibiting indicators of duplicate submissions, geographic inconsistencies, or elevated fraud-risk were flagged for review and excluded from further screening if unverifiable.

Individuals passing the prescreening stage were invited to complete a personalized eligibility assessment using REDCap (Vanderbilt University) tools hosted by University of California, Los Angeles (UCLA) [19,20]. Study staff reviewed eligibility responses and conducted telephone-based verification interviews to confirm participant identity, verify eligibility criteria, review informed consent and Health Insurance Portability and Accountability Act (HIPAA) authorization documents, and answer participant questions. During these interviews, study staff cross-checked information provided during online screening against participant self-report, resolved any discrepancies before determining final eligibility, and evaluated responses for potential inconsistencies that could indicate ineligible or fraudulent enrollment attempts. Consistent with best practices for virtual cohort studies, the research team continuously monitored recruitment data and participant-submitted documentation throughout enrollment to identify emerging patterns of fraud and other threats to data integrity, allowing participant verification procedures to be refined as needed.

### Enrollment Procedures

Eligible participants who provided informed consent were enrolled and assigned a unique study identification number. Following the completion of consent and HIPAA authorization procedures, study staff conducted a final review of eligibility documentation and approved participants for enrollment. Once approved, participants were activated within the study management database, which initiated a series of automated processes linking the participant record to a study-specific mobile app (REtain, MAintain, and SustaIN [REMAIN]). This integration enabled the secure transfer of enrollment information and established the participant’s study account, allowing immediate access to study-related activities and resources. The mobile app served as the central platform for participant engagement and study participation, including study assessments, submission of HIV viral load laboratory results, and participant reimbursement. A detailed description of the REMAIN platform features follows in the next section.

Once registered on the REMAIN app, participants completed a comprehensive baseline survey assessing factors across the domains of the Gelberg-Andersen Behavioral Model for Vulnerable Populations. Measures included predisposing factors (eg, sociodemographic characteristics, substance use, and experiences of stigma), enabling factors (eg, health care access and social support), and need-related factors (eg, diagnosed comorbidities; Figure 1). The survey took approximately 30 to 45 minutes to complete, and participants received a US $50 electronic gift card. Following survey completion, participants were asked to provide HIV viral load information using 1 of 4 approaches. Those who had undergone HIV viral load testing within 6 months of the study assessment could either provide HIPAA authorization for study staff to obtain laboratory results directly from their health care provider or upload a verifiable electronic copy of their laboratory report. Participants without a recent viral load result were offered study-supported testing through either a commercial laboratory using a study-provided requisition or at-home specimen collection using a mailed self-collection kit that was returned for laboratory analysis. Participants received an additional US $50 upon successful submission of HIV viral load information.

As enrollment progressed, participant verification procedures were continuously refined to address emerging threats to data integrity. After study staff identified attempts by some individuals to fabricate HIV laboratory reports to meet eligibility criteria, enhanced verification procedures were implemented, including manual review of all uploaded laboratory documents by trained study personnel before enrollment approval. Reviews assessed the consistency of laboratory formatting, provider information, dates, test values, and other document characteristics. It was also determined that several fraudulent laboratory reports originated from publicly available images that could be identified through internet searches for HIV laboratory results. These observations informed ongoing adaptations to the participant verification process and underscored the importance of continuous quality assurance in virtual cohort studies.

### REMAIN Mobile App

The study app was adapted from the HealthMpowerment platform, a multifeature mHealth intervention that has previously been used in HIV prevention and care studies [21-24]. The platform was customized to serve as the central hub for study participation by integrating study management (staff-facing) and mHealth functionality (participant-facing) within a single platform. Through the participant-facing app (available on Android and iOS), participants received links to study assessments, uploaded laboratory results, received study notifications and reminders, communicated with study staff, and received electronic incentive payments following completion of study activities. Through the staff-facing administrative web-based dashboard, staff could create and manage content (eg, articles, activities, and Speak Up surveys), moderate interactive features such as forums and “Ask the Expert” opportunities, communicate with participants through the messaging system, track participant milestones (surveys and test kits), and deliver incentives. Additionally, the dashboard supported extraction of participant-level data on app usage.

In addition to supporting study operations, the platform provided educational articles, HIV-related health resources, discussion forums, and engagement opportunities administered throughout follow-up. To promote participant privacy and encourage open interaction, participants selected their own usernames and engaged within the app using pseudonyms, allowing them to participate in peer-support activities while remaining anonymous to other users. Features included a peer discussion forum, an “Ask the Expert” function that connected participants with study staff and subject-matter experts, medication and daily habits tracking tools, health-related educational content, goal-setting activities, and links to external HIV and wellness resources. In addition, participants received brief “Speak Up” assessments consisting of approximately 5 to 10 questions administered at weekly intervals. These assessments captured time-varying information related to HIV medication adherence, barriers to care, mental health, substance use, and other determinants of HIV care engagement. In addition to supporting participant engagement, the REMAIN platform served as an important source of longitudinal behavioral data. The app continuously collected participant-level paradata, including the timing and frequency of logins, duration of use, and usage of all app activities [25,26].

Retention was also supported through the app using a variety of gamification strategies. Participants can earn points (“Bucks”) for engaging with app content, tracking health behaviors, and participating in staff-led challenges and engagement initiatives (eg, “Speak Up” surveys). Accumulated points can be redeemed through an in-app rewards store. App participation also unlocks avatar customization options and accessories. Additional features, including badges and reminders, were incorporated to encourage sustained engagement. Although use of the app was not required for study participation, these features were intentionally incorporated to support long-term retention and maintain participant interest throughout the follow-up period.

### Follow-Up Assessments

Participants are followed for 24 months and complete study surveys every 3 months. To balance comprehensive data collection with participant burden, survey content varies across follow-up waves. Comprehensive assessments are administered at months 6, 12, 18, and 24 and include the full battery of study measures. Assessments administered at months 3, 9, 15, and 21 consist of streamlined survey modules focused on key behavioral, psychosocial, and care engagement measures. These abbreviated assessments were designed to reduce participant burden while maintaining regular contact with participants and supporting long-term retention. HIV viral load data are collected at baseline and every 6 months thereafter. Participants received a US $50 electronic gift card for each follow-up survey, and an additional US $50 upon successful submission of HIV viral load information, which was subsequently increased to US $75 (April 2026) to improve compliance with the submission of laboratory results.

### Cohort Retention

Recognizing that participants enrolled in EPI-LoVE often face substantial social, economic, health-related, and structural challenges that can affect study participation, the cohort was designed to accommodate flexible participation. Participants were not required to complete every survey or viral load assessment to remain enrolled in the study. Individuals who missed a scheduled assessment continued to be eligible for future follow-up activities and were recontacted when subsequent assessment windows opened. This approach was intended to minimize permanent attrition, maximize opportunities for reengagement, and better reflect the realities of longitudinal participation among populations at elevated risk for disruptions in HIV care engagement.

### Ethical Considerations

The study was approved by the UCLA Institutional Review Board (IRB 23‐0477) with reliance agreements with the University of North Carolina at Chapel Hill and Florida State University. All participants provided written informed consent prior to study enrollment.

### Planned Analyses

#### Longitudinal and Predictive Analyses

Primary analyses will characterize longitudinal trajectories of HIV viral load and viral suppression by modeling viral load as a continuous outcome and viral suppression as a binary outcome. Guided by the Gelberg-Andersen Behavioral Model for Vulnerable Populations, analyses will examine how predisposing, enabling, and need-related factors, together with behavioral, structural, and digital engagement factors, influence longitudinal trajectories of viral suppression and latent class membership. Secondary analyses will evaluate movement through the HIV care continuum, with particular emphasis on transitions into and out of viral nonsuppression over time.

Longitudinal trajectory analyses will be conducted using growth mixture models (GMMs), an extension of random-effects regression that identifies distinct subgroups of individuals with similar patterns of change over time [27]. Prior to analysis, viral load distributions will be evaluated and transformed as appropriate (eg, logarithmic transformation) [28]. Viral loads will be left-censored subject to lower limits of detection. Multiple imputation of left-censored values will be conducted as a sensitivity analysis, given evidence that this approach performs comparably to likelihood-based methods that explicitly account for left-censoring [29]. We will fit GMMs using full information maximum likelihood to account for missing data under a missing at random assumption where missing data depend on observed data in the model. We will conduct sensitivity analyses by imputing data under not missing at random mechanisms to evaluate their impact on findings [30]. GMMs with increasing numbers of latent classes will be fit and compared using fit statistics (eg, Bayesian Information Criterion [BIC] and Akaike Information Criterion [AIC]), classification quality (eg, entropy), class size, model convergence, and substantive interpretability. Demographic, behavioral, psychosocial, structural, clinical, and digital factors will be evaluated as predictors of longitudinal viral load and latent class membership, with covariates selected *a priori* based on the Gelberg-Andersen conceptual model and existing literature.

Multistate Markov models will be used to characterize movement among clinically meaningful HIV care continuum states, including transitions into and out of viral suppression, while accounting for the dynamic nature of HIV care engagement [31-33]. We will start with a model that assumes that the probability of transitioning to a subsequent state depends on the participant’s current state (ie, a first-order Markov model) rather than the complete history of previous states, and that transition probabilities are constant over time (ie, time homogeneity). We will assess the appropriateness of these assumptions by relaxing them and comparing model fit to the starting model using appropriate fit statistics (eg, likelihood ratio tests) and visual diagnostics by plotting predicted and observed numbers of individuals in each state over time [34].

To support the development of personalized digital interventions, the survey and laboratory testing data will be integrated with longitudinal digital engagement measures collected through the REMAIN app to identify time-varying predictors of viral nonsuppression. Predictive models will incorporate predisposing, enabling, need-related, behavioral, structural, clinical, and digital engagement factors to identify periods of increased vulnerability and inform future delivery of tailored digital interventions. Machine learning approaches will include the least absolute shrinkage and selection operator regression, elastic net, random and fuzzy forests, and adaptive boosting to develop and validate risk prediction models for future viral nonsuppression [35-37]. Data will be randomly divided into training and test sets for the purpose of internal validation by tuning models exclusively with the training data and evaluating their performance on the test data. We anticipate using a common ratio of 70:30 for the proportion of observations in the training and test datasets but will vary the ratio, if needed, to ensure adequate sample sizes for training and model evaluation in the test data so that both datasets represent the original data [38]. For example, there may be categorical predictive factors with small counts in some categories that may lead to 0 counts if the training dataset is too small. As a sensitivity analysis, we will evaluate whether model performance improves after using the Synthetic Minority Oversampling Technique (SMOTE) to balance the distribution of viral suppression in the training data [39]. Predictive performance will be assessed using discrimination and overall prediction error that are appropriate for binary outcomes including accuracy, the F1-score, Brier score, and the area under the receiver operating characteristics curve. We will also examine visual diagnostics like the calibration and discrimination plots. We will evaluate the impact of intermittent missing data and loss-up to follow-up on model performance as a sensitivity analysis by comparing the performance of models with missing data and with missing values filled in using machine learning imputation methods [40].

#### Sample Size Calculations

The study was powered to support the primary objectives of characterizing longitudinal trajectories of HIV viral load and identifying how predisposing, enabling, and need-related factors influence changes in viral suppression over time. GMM will be used to identify distinct patterns of viral load trajectories and to examine predictors of both longitudinal viral load and latent trajectory class membership. Power calculations for these complementary analyses converged on a target sample size of approximately 1000 participants.

Sample size calculations for longitudinal viral load analyses were performed using the RMASS web-based software [41]. Assuming 5 follow-up assessments over 2 years, 70% participant retention at the final assessment, a 2-sided α=.05, and within-person correlations of 0.20‐0.30, a sample size of approximately 700 participants provides 80% power to detect small-to-moderate effect sizes (0.25‐0.30) in longitudinal viral load trajectories using random-effects regression or single-class GMM. Power is expected to be greater for continuous predictors, higher retention rates, or extended follow-up. Because analytic sample size requirements for latent class trajectory models depend on the number and distribution of underlying trajectory classes, we relied on published simulation studies to inform power for these analyses [42-44]. These studies suggest that samples of approximately 200 participants are adequate when 2 latent trajectory classes are present, whereas samples approaching 1000 participants are recommended when the underlying population comprises 4 to 5 trajectory classes. Accordingly, the planned cohort size of approximately 1000 participants provides sufficient power to support both longitudinal trajectory modeling and identification of clinically meaningful subgroups of HIV care engagement and viral suppression.

## Results

### Recruitment Outcomes

The study was funded in May 2023. Participant recruitment occurred between February 2024 and June 2025, and longitudinal data collection is ongoing with study findings anticipated in spring 2028. A total of 4789 individuals expressed interest in the study and were sent a link to the study screener, of whom 3115 initiated the screening process, and 2739 (57.2%) completed the screener (Figure 2). Among participants who completed screening, 2620 self-reported living with HIV, of whom 2262 (86.3%) met study eligibility criteria. The most common eligibility criterion was a self-reported detectable HIV viral load during the previous 12 months, reported by 1435 (63.4%) eligible participants. Among the remaining 827 eligible participants who did not report a detectable viral load in the previous year, 509 (22.5% of total eligible) qualified based on reporting a missed HIV care appointment during the past 12 months, while the remaining 318 (14.1% of total eligible) were eligible based on a qualifying mental health diagnosis. Among all eligible individuals, 1109 (49.0%) provided consent and received a study link, and 1051 subsequently completed enrollment. The majority of eligible participants were recruited through online sources (n=1749, 77.3%), while 12.9% (n=292) were referred through the clinical network and 9.8% (n=221) were referred from other research studies.

**Figure 2.**
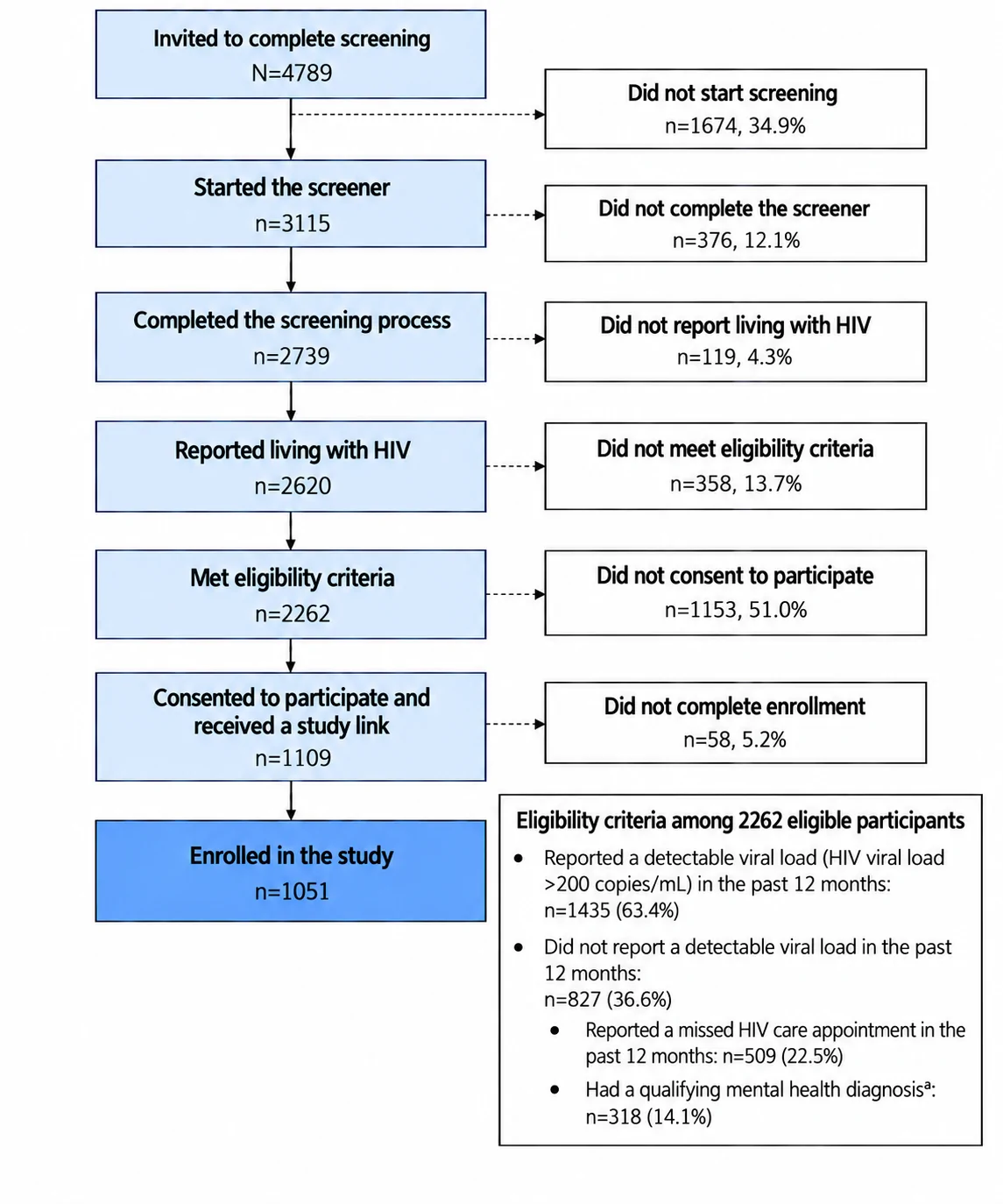
Exploring, Predicting, and Intervening on Long-Term Viral Suppression Electronically (EPI-LoVE) study recruitment and enrollment, February 2024 to June 2025. ^a^Mental health diagnoses include generalized anxiety disorder, major depressive disorder, or schizoaffective disorder.

### Baseline Characteristics

The final EPI-LoVE cohort included 1051 participants residing across 38 US states (Figure 3). Participants had a median age of 36 (IQR 31‐43) years, and most identified as male (922/1008, 91.5%; Table 1). The cohort was racially and ethnically diverse, with Black or African American participants comprising nearly two-thirds of the sample (677/1049, 64.5%), followed by Hispanic or Latinx participants (177/1049, 16.9%) and White participants (112/1049, 10.7%). Substance use was common at baseline. Overall, 77.0% (744/966) of participants reported using at least one substance during the previous 6 months, with cannabis being the most frequently reported substance (637/990, 64.3%), followed by poppers (431/1038, 41.5%) and methamphetamine (335/1009, 33.2%). Mental health symptoms were also prevalent: 39.3% (413/1051) of participants screened positive for moderate to severe anxiety (Generalized Anxiety Disorder-7 [GAD-7] score ≥10), and 63.7% (669/1051) screened positive for moderate to severe depressive symptoms (Center for Epidemiologic Studies Depression Scale-10 [CESD-10] score ≥10).

**Figure 3.**
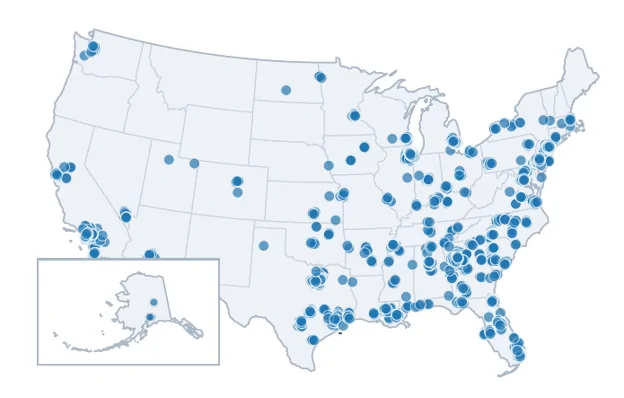
Geographical distribution of Exploring, Predicting, and Intervening on Long-Term Viral Suppression Electronically (EPI-LoVE) participants at baseline.

**Table 1.** Baseline characteristics of EPI-LoVE^a^ participants.

Characteristic	Values^b^
Age (y), median (IQR)	36 (31‐43)
Sex (N=1008), n (%)	
Male	922 (91.5)
Female	86 (8.5)
Race and ethnicity (N=1049), n (%)	
Black/African American	677 (64.5)
Hispanic/Latinx	177 (16.9)
White	112 (10.7)
Multiracial	61 (5.8)
Other	24 (2.3)
Residence^c^ (N=1051), n (%)	
Rural residence	171 (16.3)
Employment (N=1051), n (%)	
Unemployed	332 (31.6)
Mental health (N=1051), n (%)	
Moderate/severe anxiety (GAD-7^d^ ≥10)	413 (39.3)
Moderate/severe depressive symptoms (CESD-10^e^ ≥10)	669 (63.7)
Substance use, past 6 months, n/N (%)	
Any substance use	744/966 (77.0)
Cannabis	637/990 (64.3)
Cocaine	243/1043 (23.3)
Ecstasy	195/1043 (18.7)
Fentanyl	44/1044 (4.2)
Heroin	40/1043 (3.8)
Methamphetamine	335/1009 (33.2)
Poppers	431/1038 (41.5)
Prescription opioids	114/1044 (10.9)
HIV characteristics, n/N (%)	
Missed ≥1 HIV care appointment, past 6 months	592/986 (60.0)
HIV RNA level ≥200 copies/mL	315/851 (37.0)

a EPI-LoVE: Exploring, Predicting, and Intervening on Long-Term Viral Suppression Electronically.

b The sum may not equal the total due to missing data.

c Rural residence was defined based on whether participants’ current residence met the rural designation of at least one of three federal classification systems: the Federal Office of Rural Health Policy (FORHP), Ending the HIV Epidemic (EHE), or the National Center for Health Statistics (NCHS).

d GAD-7: Generalized Anxiety Disorder 7 items scale.

e CESD-10: Center for Epidemiologic Studies Depression 10 items scale.

By design, the cohort reflects individuals experiencing challenges across the HIV care continuum rather than solely those who were not virally suppressed at enrollment. Among the 1051 enrolled participants, 863 (82.1%) successfully submitted a baseline HIV viral load result. Participants were offered multiple options for providing viral load data, allowing them to select the approach that best fit their circumstances. The most common method among participants who submitted HIV viral load results was self-upload of an existing laboratory report (355/863, 41.1%), followed by at-home self-collection using a mailed testing kit (211/863, 24.4%), provider submission following HIPAA authorization (187/863, 21.7%), and study-facilitated testing through a commercial laboratory (110/863, 12.7%). It should be noted that completion rates varied by submission method. Provider-facilitated retrieval of laboratory results following HIPAA authorization yielded the highest completion rate, with 96.4% (187/194) of viral load data successfully retrieved from the provider. Participants who chose to upload their own laboratory reports also demonstrated high follow-through (355/408, 87.0%). Completion was lower among participants who selected at-home self-collection, with 70.3% (211/300) returning a completed specimen for testing.

Among participants with a baseline viral load, 62.7% (541/863) had an HIV viral load <200 copies/mL but met study eligibility criteria because of prior viral nonsuppression or elevated risk for future viral nonsuppression, including having missed one or more HIV care appointments during the previous 6 months (592/986, 60.0%). The remaining 37.0% (315/851) were not virally suppressed (HIV viral load >200 copies/mL) at enrollment.

## Discussion

### Principal Findings

The EPI-LoVE study demonstrates the feasibility of establishing a national virtual cohort of people living with HIV who are at elevated risk of viral nonsuppression. Through a combination of digital recruitment strategies, clinic-based partnerships, and participant-centered engagement approaches, EPI-LoVE successfully enrolled a geographically diverse cohort of more than 1000 participants from 38 US states. The study further demonstrates the feasibility of integrating longitudinal behavioral assessments, HIV viral load biomarkers, app-based engagement metrics, and high-frequency participant check-ins into a fully remote research platform, providing a scalable model for decentralized HIV cohort studies. EPI-LoVE was specifically designed to address important limitations of traditional clinic-based HIV cohorts, which often underrepresent individuals experiencing disruptions in care, housing instability, substance use, mental health conditions, geographic barriers, and other factors associated with suboptimal HIV outcomes. By reducing the need for in-person participation, the virtual cohort model expanded geographic reach, reduced participant burden, and facilitated enrollment of populations that are frequently underrepresented in longitudinal HIV research.

### Lessons Learned From Conducting a National Virtual Cohort

The EPI-LoVE experience demonstrated that successful virtual cohort recruitment requires not only well-defined enrollment procedures but also the ability to rapidly adapt recruitment and verification strategies as new challenges emerge. Although the study initially used standard procedures commonly used in virtual research, ongoing monitoring of recruitment performance, participant behavior, and data quality necessitated continuous refinement of recruitment and quality assurance processes. As recruitment progressed, the study team identified attempts by some individuals to submit fabricated HIV laboratory reports to establish study eligibility. In response, enhanced verification procedures were implemented, including the manual review of uploaded laboratory documents and real-time quality control checks before participants progressed from screening to enrollment. In several instances, fraudulent laboratory reports were traced to publicly available images obtained via internet searches. This observation highlighted the ease with which digital documentation can be manipulated and underscored the importance of ongoing quality control procedures in virtual research environments.

Another key lesson is that no single approach to remote HIV viral load ascertainment was sufficient to meet the needs of a geographically diverse national cohort. Participants were offered multiple options for submitting viral load data, each with distinct strengths and limitations. Retrieval of laboratory results through HIPAA authorization achieved the highest completion rates because it minimized participant burden. However, this approach depended on participants remaining engaged in HIV care and receiving routine laboratory monitoring, making it less practical for a cohort intentionally designed to include individuals at risk for disruptions in care. Participant upload of existing laboratory reports provided an effective alternative but required robust verification procedures to ensure data integrity.

For participants requiring new laboratory testing, both commercial laboratory testing and at-home self-collection presented implementation challenges. Travel distance and inconvenient laboratory locations were common barriers to completion at commercial laboratories, particularly for participants relying on public transportation. Although staff frequently assisted with transportation planning and identifying alternative testing locations, geographic barriers remained for some participants. At-home self-collection eliminated travel but introduced different challenges, including participant difficulty completing specimen collection, misplaced kits, and the need to distribute replacement kits. Because repeated kit replacement was costly, limits were ultimately placed on the number of replacement kits provided for each testing interval. Ultimately, enhancing participant support, strengthening verification procedures, and increasing participant compensation for laboratory completion all contributed to improved biomarker ascertainment as the study progressed. Importantly, we note that the “best” laboratory collection strategy varied across participants, reinforcing that flexibility rather than standardization may be the key to successful remote biomarker collection.

### Strengths and Limitations

Several features distinguish EPI-LoVE from previous HIV cohort studies. First, the study intentionally focused on individuals who were currently not virally suppressed, had a history of viral nonsuppression, or possessed characteristics associated with future viral nonsuppression. While many HIV cohorts are comprised primarily of individuals already engaged in care, EPI-LoVE was designed to better represent those experiencing challenges across the HIV care continuum. Second, EPI-LoVE integrated multiple sources of data, including repeated survey assessments, HIV viral load measurements, and detailed app-based engagement metrics. The combination of traditional epidemiologic measures with digital behavioral data provides a unique opportunity to better understand the dynamic factors associated with HIV care engagement and viral suppression over time. Third, the REMAIN mobile app served not only as a retention tool but also as a mechanism for data collection, participant engagement, health education, and potential intervention delivery. The integration of these functions within a single platform may provide a scalable model for future HIV cohort studies and digital interventions.

Several limitations should also be acknowledged. Participation required access to a smartphone and sufficient digital literacy to engage with study procedures, potentially limiting representation among some populations. Although extensive verification procedures were implemented, virtual studies remain vulnerable to fraudulent enrollment attempts and require ongoing quality assurance efforts [45,46]. In addition, participants were not recruited using probability-based sampling methods; therefore, findings are not generalizable to all people living with HIV in the United States. Finally, app engagement was voluntary, and varying levels of engagement may influence the completeness of some digital measures.

### Conclusions

EPI-LoVE demonstrates the feasibility of establishing and engaging a large national virtual cohort of people living with HIV who are experiencing or are at elevated risk of viral nonsuppression. By integrating remote recruitment, biomarker collection, repeated behavioral assessments, and high-frequency digital engagement data within a single platform, the study provides a scalable framework for decentralized HIV cohort research. Beyond establishing the cohort itself, EPI-LoVE offers practical lessons regarding participant recruitment, verification, remote biomarker collection, and retention that may inform the design of future virtual studies. As digital health technologies and predictive analytics continue to evolve, platforms such as EPI-LoVE have the potential to identify individuals at increased risk for poor HIV outcomes in near real time and support delivery of personalized, just-in-time digital interventions. More broadly, the methodological approaches developed through EPI-LoVE may also be applicable to virtual cohort studies of other chronic health conditions that require long-term monitoring, sustained engagement, and integration of behavioral, clinical, and digital data.

## Supplementary material

10.2196/108764 Peer Review Report 1 Peer review report by the National Institute of Allergy and Infectious Diseases (NIH/NIAID).

## Abbreviations

AIC: Akaike Information Criterion
BIC: Bayesian Information Criterion
CESD-10: Center for Epidemiologic Studies Depression Scale-10
EPI-LoVE: Exploring, Predicting, and Intervening on Long-Term Viral Suppression Electronically
GAD-7: Generalized Anxiety Disorder-7
GMM: growth mixture model
HIPAA: Health Insurance Portability and Accountability Act
mHealth: mobile health
REMAIN: REtain, MAintain, and SustaIN
SMOTE: Synthetic Minority Oversampling Technique
UCLA: University of California, Los Angeles
