# Supplementary material for: A US-Based National Virtual Cohort of People Living With HIV at Risk for Viral Nonsuppression: Protocol for the EPI-LoVE Prospective Cohort Study
Source: JMIR Res Protoc. 2026 Oct 7;15:e108764. doi: 10.2196/108764 (PMC13645198; doi:10.2196/108764)
Supplement: Supplementary file 1 [file resprot-v15-e108764-s001.pdf]

1UG3AI176592-01 Gorbach, P

RESUME AND SUMMARY OF DISCUSSION: This very good new Exploratory/Developmental Phased Award Cooperative Agreement application entitled “Exploring, Predicting, and Intervening on Long-term Viral suppression Electronically (EPI-LoVE)” was submitted in response to AI22-024: Limited Interaction Targeted Epidemiology: Viral Suppression (LITE-VS) (UG3/UH3 CLINICAL TRIAL OPTIONAL) by the University Of California Los Angeles, Los Angeles, California with Dr. Pamina Gorbach as Program Director/Principal Investigator (PD/PI).

The review panel identified several strengths in this application. The application proposes to use evidence-based methods for digital recruitment, retention, and interventions. This includes venue-based recruitment from a large network of HIV care sites, advanced digital advertising methods, and an existing app platform that the team has developed and used successfully in prior cohort studies. The PI and team have plenty experience establishing digital cohorts using the proposed methods and has access to scientific environments with extensive research-related resources. Describing milestones for high-priority subgroups was an additional strength. The transition milestones would ensure adequate recruitment overall and within key subgroups, including people who are Black, rural, young, or using substances. Overall, the cohort plans generally align well with the goals of the RFA, the team is experienced and well-rounded, and the environment is excellent and capable of supporting the work.

However, a number of weaknesses also raise concerns about the feasibility of the plan and the impact that the proposed analyses would have on the field. Some of weaknesses include lack of innovation in the proposed methods, most of which have been applied in the investigators’ prior cohort studies; lack of a transition milestone for enrollment of participants with unsuppressed HIV; and lack of details on threshold selection or evaluation of fairness in the risk prediction model.

DESCRIPTION (provided by applicant): In response to RFA-AI22-024 Limited Interaction Targeted Epidemiology: Viral Suppression (LITE-VS), we propose “Exploring, Predicting, and Intervening on Long-term Viral suppression Electronically (EPI-LoVE)” with a cross-cutting, interdisciplinary scientific team with strong expertise in longitudinal cohorts to enroll a large, digitally maintained cohort of people living with HIV (PLWH) in the US who are not adequately virally suppressed (>200 copies/mL), report prior gaps in HIV care engagement, or lack of sustained viral suppression (VS). The cohort will investigate trajectories of HIV care engagement and VS applying a theoretical approach and analytic strategies that recognize the cyclical nature of retention in HIV care and VS. We will examine the syndemics underlying these outcomes including substance use, mental health, and barriers to HIV care including stigma and rural isolation. Our strategy includes multiple recruitment methods such as a) social media recruitment in partnership with digital advertising companies employing unique tools, websites/apps and artificial intelligence (AI)-based models to optimize outreach/recruitment; and b) a partnership with AIDS Healthcare Foundation (AHF), the largest HIV medical provider in the country. We will enroll a diverse cohort of PLWH enriched with those living in rural areas, Black, under 25 years of age and experiencing substance use challenges. An evidence-based digital platform (HealthMpowerment, HMP) deployed in 10 studies, including other limited interaction targeted epidemiology (LITE) initiatives, will be used for data collection, real-time analysis and participant communication and will be supplemented with semi-annual blood specimens collected with a multi-pronged strategy (i.e. local laboratory, clinics, and home-

based collection options) to facilitate participation. We will develop a sophisticated risk assessment tool to optimize when PLWH in our cohort are most at risk for VNS and deliver tailored, real-time intervention components based on their personalized needs at the time they need them most. Our investigative team from three leading institutions The University of California at Los Angeles and Irvine, and University of North Carolina, Chapel Hill has decades of experience with recruitment, engagement, and care of PLHW and large-scale longitudinal cohort studies, as well as long-standing expertise developing digital and AI-based tools in collaboration with HIV stakeholders. We capitalize upon productive partnerships and expertise to articulate the drivers of the ongoing HIV epidemic among the most vulnerable populations in the US and to identify the most effective, expeditious and scalable digital strategies to address this ongoing public health crisis.

PUBLIC HEALTH RELEVANCE: In response to RFA-AI22-024 we propose “Exploring, Predicting, and Intervening on Long-term Viral suppression Electronically (EPI-LoVE)” to enroll, retain, analyze HIV care outcomes, and test digital health interventions among a large diverse digital cohort of people living with HIV (PLWH) who are struggling to engage with care and maintain consistent viral suppression. With multiple recruitment methods including social media and AIDS Healthcare Foundation current and former patients, we employ an evidence-based digital platform (HealthMpowerment, HMP) and multiple data sources (e.g., surveys, biologic specimens, app data). Capitalizing upon cross- cutting expertise to articulate the drivers of the ongoing HIV epidemic among the most vulnerable populations in the US we will identify the digital strategies that address the intersectionality of this ongoing public health crisis.

CRITIQUE: The comments in the CRITIQUE section were prepared by the reviewers assigned to this application and are provided without significant modification or editing by staff. They are included to indicate the range of comments made during the discussion and may not reflect the final outcome. The RESUME AND SUMMARY OF DISCUSSION section summarizes the final opinion of the committee after the discussion and is the basis for the assigned Overall Impact/Priority score.

CRITIQUE 1

Significance:	2
Investigator(s):	2
Innovation:	2
Approach:	4
Environment:	1

Overall Impact: This exceptional team of qualified MPIs has crafted a compelling approach to investigate trajectories of HIV care engagement and VS by applying a theoretical approach and analytic strategies that recognize the cyclical nature of retention in HIV care and VS and applies a previously established App that would need to be further tailored/tested for this purpose. They will examine the syndemics underlying these outcomes including substance use, mental health, and barriers to HIV care including stigma and rural isolation. The overall approach is ambitious and quite complex and leaves open the possibility for a number of potential challenges to success, as mentioned in the Approach. As described, the MPI arrangement allows a range of specific expertise but seems more complex than optimal although these investigators have substantial experience collaborating. The team clearly describes how this effort could address many of the common barriers to care and follow up and has the potential to demonstrate the practical impact of intervening in this manner across real world care settings using an adapted web-based platform that would allow intervention to occur at precisely the right time and manner to ensure VS among a much greater segment of a key risk population. Even with the noted

practical concerns, primarily regarding the approach, this is an impressive application that has solid potential to impact the field as well as care settings.

1. Significance:

Strengths

- This team targets key drivers of the ongoing HIV epidemic among the most vulnerable populations in the US with the aim of establishing the most effective, expeditious and scalable digital strategies to address this ongoing public health crisis.
- They will implement multiple recruitment methods including a) social media recruitment in partnership with digital advertising companies employing unique tools, websites/apps and artificial intelligence (AI)-based models to optimize outreach/recruitment; and b) a partnership with one of the largest HIV medical provider in the country.
- The team lays out a compelling approach to investigate trajectories of HIV care engagement and VS by applying a theoretical approach and analytic strategies that recognize the cyclical nature of retention in HIV care and VS.
- They will examine the syndemics underlying these outcomes including substance use, mental health, and barriers to HIV care including stigma and rural isolation. This combination of outcomes has not been adequately examined and doing so successfully has the potential for moving the field forward.

Weaknesses

- The team seeks to address a critical and complex problem with a project that also appears quite complex and ambitious. Several of the practical issues mentioned in the Approach may limit the impact of the outcomes even if successful.

2. Investigators:

Strengths

- The MPIs all have substantial experience in the field, including leadership of large trials across the US, and particularly in the southern areas of interest.
- The MPI arrangement with Drs. Gorbach, Hightow-Weidman, & Young seems reasonable and well-conceived in terms of the division of expertise, decision-making, and ability to oversee complex data analytical designs stemming from web-based work.
- The leaders and team members have the required expertise with web-based data collection and App development/use, and this will be crucial in the proposed work.
- The team has a clear track record collaborating on papers and other relevant work in the field, which boosts confidence that they can be successful with the proposed project.

Weaknesses

- Though the MPI arrangement makes sense in terms of each person bringing specific expertise to the overall project, it is not clear that having three PIs is necessary and seems a bit more complex than optimal.

3. Innovation:

Strengths

- Driven by gaps in the field, this team seeks to examine syndemics underlying challenges to VS outcomes including substance use, mental health, and barriers to HIV care including stigma and rural isolation.
- Very few approaches have successfully intervened on challenges to VS using individually tailored interventions as well as tailored outcome measures (e.g., DBS kits if preferred).
- Many of the barriers to care and follow up are being addressed by the proposed work and this has the potential to demonstrate the practical impact of intervening in this manner across real world care settings using a web-based platform.

Weaknesses

- The use of web-based approaches are not innovative in themselves but do have the potential to remove a number of barriers to care and follow up although this will admittedly reduce the intervention reach by requiring this critical patient population to have ongoing access to smart phones.

4. Approach:

Strengths

- The HMP app has been developed and tested and would be optimized during this study and tailored to the individual.
- The concept of individualized intervention will be pushed to the limits in this innovative approach and could be an important step forward for adherence-focused intervention work among high-risk populations.
- This team makes every effort to alleviate many of the barriers in reaching the key demographics within PLWH and even tailors the use of various outcome measures to accommodate the individual (e.g., use of DBS kits).
- More than sufficient expertise will be available to conduct complex data analysis within an ambitious research framework.

Weaknesses

- Recruitment seems to be a challenge and an area that is less clear although the team has anticipated ways to improve it if needed (expanding sites within the AHF network).
- They report very precise characteristics of their expected sample, which will be ideal if that is achieved but it is unclear exactly if/how they plan to e.g., oversample certain demographics to meet those characteristics. It is simply stated that those will be the characteristics they're after but not how recruitment will be managed if/when certain demographics are more difficult to reach.
- Admittedly, including only those with a smartphone will further restrict recruitment. In combination with other severely limiting factors, as they report from prior work (~23% of sampled PLWH needing VS intervention) may challenge feasibility although they do indicate their plan to potentially expand recruitment to other AHF clinics in the network if needed.
- Developing a risk score is one of the aims that will be based on the study data (TBD), so there is no preliminary indication about how this will work or how it will impact the eventual development of the tailored intervention in the final aim.

- Because so many different sites will be included in the recruitment reach, it is likely that this will complicate some of the outcome analyses in unanticipated ways, generalizability of findings, and will potentially introduce a number of confounds to the data set. The team understands this as they have substantial experience with web-based data collection and intervention research, which is encouraging to a degree.
- They plan to provide the option for using multiple types of data sources for the same outcome (biological, self-report, DBS kits), which makes it more flexible for participants and fully aligns with the 'no barriers' approach but could also introduce unpredictable variation in the data across participants and potential differences across sites/regions.

5. Environment:

Strengths

- This research will be conducted in the context of exceptional institutions with the resources and experience necessary for success. Key personnel will have more than adequate support for the range of activities being proposed.
- MPIs will bring to bear resources from UCLA, UNC, UC-Irvine, and UNC as well as associated clinical sites through one of the largest AIDS healthcare networks (AHF). Their familiarity with the proposed sites as well as existing collaborations through other grant-funded efforts will provided strong support to the studies being proposed.
- The leaders of this work have an organized plan for recruitment, data collection, and web-based intervention testing in the context of primarily web-based efforts but also have flexible plans that include interfacing with individual clinical sites if needed.
- The plan is to expand clinical sites, which are also within the AIDS healthcare foundation network, if they deem it necessary for greater recruitment over time.

Weaknesses

- This research will include a complex network of institutions/sites/leadership, though feasible, it is not clear that this is necessary for the proposed efforts.

Study Timeline:
ACCEPTABLE
Comments or Concerns: Timeline was included and appears reasonable given what's described in the application.

CRITIQUE 2

Significance:	4
Investigator(s):	2
Innovation:	3
Approach:	4
Environment:	2

Overall Impact: The proposed study, EPI-LoVE, seeks to enroll a national cohort of people living with HIV (PLHIV) who are not virally suppressed or who have a history of disengagement in HIV care. Data collected through the cohort will be used to identify longitudinal patterns of care engagement and viral suppression, develop and validate a risk prediction tool, and trial digital interventions for people at high risk of viral non-suppression. The application is led by a strong multi-disciplinary collaborative team with expertise in HIV-related cohort studies, digital health interventions, artificial intelligence and machine learning, and statistical analysis and development of risk prediction methods. This team is further supported by an IT consulting firm with a long-standing relationship with the investigators to develop the HMP app and a collaboration with AIDS Healthcare Foundation, of which the clinical networks will serve as a recruitment source for the cohort. These strengths are weighed against the unfocused nature of this application and doubt about the feasibility in accomplishing these aims within the 5 years of the study.

1. Significance:

Strengths

- This study is poised to answer important questions about trajectories and predictors of viral load suppression (or non-suppression).
- The use of multiple strategies for recruitment and viral load testing, while probably not intended as such, will be informative to understanding more about optimal methods (i.e., acceptability, efficiency, potential for bias, etc.) for future digital research with PLHIV and subpopulations.
- Some recent trials with PLHIV have experienced difficulty enrolling people who are not virally suppressed, given that these trials tend to reach people within clinical catchment areas and who are thus typically engaged in care. This may be a gap that digital cohorts could address. Therefore, demonstrated feasibility of this cohort to enroll PLHIV who are not virally suppressed or at risk of non-suppression will be a methodologic advancement in terms of strategies to reach, engage, and provide interventions to support engagement in care and treatment adherence.

Weaknesses

- Despite the potential methodologic advances and the wealth of data collected, the investigators do not have a plan to evaluate the efficiencies and potential biases of these various recruitment, survey (quarterly surveys vs check-ins), and blood collection strategies, as well as factors associated with attrition. Further, there is so much that can be scientifically explored to evaluate whether digital cohorts (broadly) and the specific recruitment/retention, methods of measurement and data collection, utilized within such cohorts can overcome current limitations of traditional (in-person) cohorts. There is no apparent analysis for Aim 1.
- Data could be utilized to answer many questions about viral suppression among PLHIV in the US to advance EHE goals, but there are no further proposed analysis of the observational data beyond the trajectory analysis and no clear description of how the HMP/digital health interventions (DHI) or risk assessment tool could be utilized or scale-up outside of this study.
- There is significant focus on the app and DHIs but little evidence provided to support the use of an app to address gaps in the HIV care continuum.

2. Investigators:

Strengths

- This is a strong interdisciplinary scientific team with strong expertise in longitudinal cohorts, electronic research methods, data management and harmonization, statistical and epidemiologic methods including modeling, digital health intervention design and implementation, development and evaluation, and HIV prevention and treatment.
- Dr. Gorbach has extensive experience in assembling and maintaining large clinical cohorts, including with PLHIV (e.g., CP3NO, mSTUDY).
- Dr. Young (mPI) has expertise in digital interventions, AI, and big data and evaluated ethical considerations and participant perspectives in these methods.
- Dr. Javanbakht – expertise in cohort studies, longitudinal and trajectory analyses.
- Expertise in modeling, machine learning, and informatic by Drs. Comulada and Bui.
- Community input by UCLA's and UNC's CABs.
- Digital health intervention expertise by Drs. Hightow-Weidman and Muessig.
- Dr. Rosenberg has expertise in developing risk assessment tools using AI and machine learning techniques, one being validated among 13 nationally representative datasets in sub-Saharan Africa.
- Dr. Gorbach has expertise in implementation science and social epidemiology, cohorts and RCTs with various key populations, and experience implementing a UG3/UH3.

Weaknesses

- The study seems quite top-heavy with many senior investigators and only two coordinators (one at UNC and one at UCLA) at 6cal/mo for a study of this scale and complexity. It will be helpful to provide options to participants for blood collection and utilize multiple strategies for recruitment, but that also comes with added complexity for implementation.

3. Innovation:

Strengths

- The trajectory analyses to model time in different states of viral suppression is a thoughtful and innovative approach to identifying subgroup patterns of VNS and predictors of VNS.
- The investigators propose to use a risk assessment tool, which will be a data-driven approach to optimize intervention delivery when at risk.

Weaknesses

- Partnering with a social media and advertising agency and use of the HMP digital platform for this study is not particularly innovative.
- Appreciating that the app is available on Android and iOS and optimized for low bandwidth/connectivity (meaning available offline?), there are still questions about equity and who may be effectively reached and retained if the inclusion criteria require personal access to a smartphone device and overall heavy reliance on an app. (Smartphone access is not a requirement of the RFA.)
- There is a wealth of data demonstrating the role of social and structural determinants in HIV outcomes and those same determinants of health also align with consistent technology access, but that is ignored in this study design. For example, past publications from a prior HMP trial compared the app compliers (n=50) to non-compliers (n=144); non-compliers were less likely to have insurance, college education, and (though not statistically different) recent homelessness

and arrest. This raises questions about whether people are “non-compliant” because of access issues and other life priorities. Further, if digital research excludes people who are do not have access to smartphones and fails to retain people with intermittent access, is it excluding those who are most vulnerable to worse HIV outcomes? Unfortunately, it is not acknowledged and there are no apparent plans measure or evaluate these questions in the application.

4. Approach:

Strengths

- Recruitment strategy will draw on collaborations with a network of HIV service organizations (AIDS Healthcare Foundation), collaboration with a digital advertising company, and partnerships with existing clinical research cohorts. This increases the likelihood that the investigators achieve their target enrollment and their objective of enrolling a sample with diversity in geography, race, and ethnicity.
- I had initial concerns about the use of DBS or TASSO (based on their return rates for the HMP RCT), but note the use of multiple options (LabCorp, AHF facility, TASSO, and willingness to accept recent lab result), which will increase the likelihood of receiving VL results.
- The study team utilizes paradata, which does not rely on self-report, to evaluate use of the app.
- Growth mixture modeling, as well as Markov Chain modeling, will be utilized to explore dynamic trajectories and subgroups across the HIV care continuum over time.

Weaknesses

- The research team proposes to collect a range of data pertaining to their conceptual model; however, there is no collection of internet/technology access (including consistency of access), digital health literacy, and general internet/technology use, all of which would also align with the domains of the conceptual model. These data are important to understanding whether technology is associated with attrition and HIV outcomes, not to mention important for informing future interventions that rely on digital technology.
- The AI recruitment approach is interesting and may increase the speed of recruitment; however, there is no description of how the ‘look alike modeling’ (based on identifying similar people to those who clicked on an add and enrolled) will be paced to 1) avoid fraudulent enrollment (e.g. targeting sites in which people share information about incentivized studies and eligibility requirements) and 2) limit bias (e.g. rapid enrollment of non-Hispanic White participants).
- To the point above, the study has several validation checks in place, but they describe that the system is automated to provide the study incentive as soon as an individual is enrolled and completes the baseline survey. While the validation listed may prevent duplicate enrollments, people from outside the eligible geographic region from enrolling, it is not likely to catch people who falsely respond to screening questions and take a survey for a quick incentive. Installation of a study app will likely serve as a barrier for some, but certainly not all fraudulent participants. What will eventually screen out most fraudulent participants is the viral load testing, which will also screen out PLHIV who are not interested or capable of completing that component. However, the study automatically provides a $30 incentive for completion of the survey. Based on experiences of other digital cohorts, there will likely be hundreds or thousands of people who complete the screening and baseline survey (and to whom TASSOs are shipped) but who are not ultimately enrolled (based on VL testing) and followed in the cohort. This is not planned for in terms of time, budget, and human resources.

- The Go/No Go Transition Milestones do not include any milestones based on viral suppression, as specified in the RFA.
- Unclear how other HIV care continuum data, pertinent to Aim 2 and 3, will be collected and operationalized. In some sections it appears that HIV care and treatment adherence are measured through daily/weekly app-based check-ins. This is vague with respect to outcome measures and there is no evidence provided to suggest that a cohort will respond to daily/weekly check-ins for the duration of follow-up.
- Unclear how long participants will be followed – timeline indicates 18 months, protocol synopsis indicates 48 months, and Subject Participant Duration in the Protocol Synopsis indicates 36-48 months – and then whether there would be sufficient data and time on study to achieve these aims.
- The DHI seems to include a mix of interventions, but they are not well described, and it is unclear how these will be assigned based on risk.
- The timeline seems tight. The investigators do appropriately plan for approximately 21 months of recruitment and indicate that this time could be extended for another 3 months, if necessary. The expected of # of participants per month is underestimated- this would be at least 42 participants per month, not 23, if only 1,000 people are preliminarily enrolled in the baseline survey, but likely to be much more than 1,000 who complete the survey. This could impact the time to develop the risk assessment tool and the feasibility of achieving aim 3. There are other components in the timeline, including qualitative research, EBT and TMI, that are not described but may also impact progress to complete the 3rd study aim.
- Overall, this application seems unfocused in that it brings together leading investigators in these areas, but the methods and objects are not well integrated.

5. Environment:

Strengths

- The scientific environment and complementary collaborations with IT agencies and a national HIV care provider increase the probability of success of this study in achieving their proposed aims.
- The lead institutions, UCLA, UC Irvine, and UNC provide the infrastructure and resources necessary for cohort implementation, AI and machine learning, and development of the app and DHI, respectively.

Weaknesses

- Based on the budget justifications, there does not seem to be a site identified as responsible for the logistics of cohort implementation, e.g. trouble-shooting participant issues, managing incentives, coordinating shipping (and covering costs) of TASSO devices and other blood collection/testing. While much can be automated, there will still be significant logistics and troubleshooting that will need to be managed.

Study Timeline:
UNACCEPTABLE
Comments or Concerns: There are elements introduced in this section that are not addressed elsewhere, including involvement of the CDC, qualitative methods, and testing of TMI and EBT(?). It also underestimates the monthly enrollment based on the sample size and estimated duration of enrollment.

CRITIQUE 3

Significance:	4
Investigator(s):	2
Innovation:	6
Approach:	6
Environment:	1

Overall Impact: This application from investigators at UCLA, University of North Carolina, and the University of California – Irvine proposes to use digital marketing on various websites and partnerships with a commercial advertising agency (Csphere) and a large healthcare organization (AIDS Healthcare Foundation) to recruit 1,000 individuals who are not virally suppressed, are not engaged in care, or who have other risk factors (substance use or mental health disorder). They would then follow these participants for up to 48 months, using a smartphone app the team developed (HealthMPowerment) to collect quarterly online surveys, conduct "daily/weekly check-ins," and facilitate viral load testing every 6 months. The team then proposes to conduct analyses exploring trajectories of care engagement and will deliver interventions through the app intended to increase rates of viral suppression. Overall, the cohort plans generally align well with the goals of the RFA, the team is experienced and well-rounded, and the environment is excellent and capable of supporting the work. However, a number of weaknesses also raise important concerns about the feasibility of the plan and the impact that the proposed analyses would have on the field. The most important concern is that very few details are provided about how the team will uniquely address what is likely to be the most challenging part of this work: Finding and enrolling individuals who have unsuppressed virus. Most of the description of this work involves vague references to methods (e.g., "AI-based tools," "machine learning") with very few specifics, raising doubts that the team has a sound plan to meet the challenge. Few of the methods proposed in this work are truly innovative, and it is not particularly clear exactly how the proposed analyses will advance the science about factors involved in care disengagement/viral non-suppression. The project also seems to include providing interventions to enrolled participants, but no methods are described for evaluating the effects of these interventions on clinically relevant outcomes or, if useful, how these interventions might benefit patients in the real world.

1. Significance:

Strengths

- The project aims to recruit and retain individuals living with HIV but who are not virally suppressed, who are among the highest-priority populations for HIV prevention.
- Analyses focused on transitions across the HIV care continuum could be informative.

Weaknesses

- Even if the aims are achieved, it is not clear how scientific knowledge, technical capability, or clinical practice would be improved.

2. Investigators:

Strengths

- Dr. Gorbach has expertise recruiting large cohorts of those at risk for HIV and substance abuse.
- Dr. Hightow-Wideman has considerable expertise developing technology-assisted interventions for HIV and HIV research in general.

- The team is well-rounded, and includes epidemiologists, statisticians, informaticians, implementation scientists, behavioral scientists.

Weaknesses

- Although Dr. Gorbach has led large cohort studies in the past, many of these have been largely locally oriented and their scientific impact is not clear.
- The roles of many investigators not clear, including what specifically each investigator will do to advance the proposed aims. Dr. Bui's biosketch also appears to describe his role on a NIDA-funded cohort project.
- It appears that Dr. Young will be primarily in charge of recruiting the cohort, and while he has expertise in somewhat similar work, including predicting disease trends from social media data, it is not clear that he has recruited large cohorts of individuals who are not virally suppressed.

3. Innovation:

Strengths

- Using a smartphone-based platform to help participants stay engaged over time is an interesting strategy, and the team appropriately pairs this with emails, SMS, and calls outside the app.

Weaknesses

- Contracting with advertising companies to recruit participants on social media is not innovative.
- The proposed smartphone application has been the focus of 10 previous studies.
- Although listed as an innovation, collecting data in a variety of different ways is not particularly novel.
- Risk assessments are not novel, and it is not clear how it would be used to identify when individuals at risk or what intervention content they would be provided.

4. Approach:

Strengths

- Supplementing online recruitment with a partnership with AIDS Healthcare Foundation (AHF) could be more effective than relying solely on either approach alone.
- Thinking about the "user experience" of engaging in cohort-focused research is a unique perspective that could yield improvements in retention.
- The proposed smartphone app has features that could improve retention among participants.
- The team has had success recruiting relatively large samples with sizable percentages of Black and racial/ethnic minority individuals.
- Enabling participants to submit viral load data in multiple ways is a significant strength that could increase adherence rates.
- The team's approach to modeling participants' transition through various stages of the HIV care continuum are fitting and could yield useful findings.

Weaknesses

- The application contains very few details about what exactly the team will do to accomplish what is likely to be a very challenging task: Finding and recruiting 1,000 people who are not virally suppressed. Aside from the usual techniques (online advertising), investigators suggest they will

rely on commercial partners that have extensive consumer databases with “large-scale … social/behavioral datasets,” but it is not clear how/whether this will be useful in recruiting individuals with HIV who have detectable viral loads, specifically.

- Investigators also suggest that they have used “AI..based models” to identify HIV-relevant influencers, but the work cited on this used only Twitter data (one of the only social media platforms that provides public access to user content), identified only 23 influencers, and does not appear to have explored whether these influencers could help reach individuals with unsuppressed virus. Arguing that this will certainly be a fruitful approach seems like an overstatement.
- There is very little information about which specific geographic regions the team will recruit from and why.
- Although partnering with AHF to supplement digital recruitment is likely a strength, the team suggests that AHF already “routinely utilizes out of care lists and care navigators” to try to re-engage unsuppressed individuals in care. Few details are provided about what the team will do that would be more successful in engaging these individuals that AHF has not already done and raise doubts about whether this avenue will be beneficial for recruitment.
- Cohort eligibility criteria are not clear and would seem to risk potentially recruiting many individuals who are functionally virally suppressed. For example, while one criterion is “not currently virally suppressed,” no specific threshold is included, and it is not clear how the team will assess this criterion at enrollment. The team also suggests that participants who have HIV and who have substance use disorders or mental health conditions would also be eligible, but many of these individuals are likely to be virally suppressed, so the rationale for including this criterion is not clear.
- Although the team plans to verify the viral loads of all enrolled participants within the first 3 months, and this seems likely to be effective in validating that all participants have HIV and their viral loads, there are no other steps proposed to validate participants being recruited digitally upon enrollment, other than simple fraud-mitigation strategies that can be easily overcome. This could lead to considerable waste in expensive blood sampling and analysis on fraudulent enrollments that could limit the budget available for recruiting valid participants. It is also unclear what “confirmation of IP addresses, zip code, and latitude/longitude” means.
- While developing risk assessment tools may be useful, several such tools exist (and, to my understanding, have received limited use in the real world so far). It is not clear how the proposed tool would improve upon these or how the proposed tool would realize more real-world use. Very few details provided about it, as well.
- The rationale for and focus of daily/weekly app-based “check-ins” is not clear and seem likely to risk leading participants to disengage from demanding too much of them (notification fatigue, etc.).
- It seems some of the app components that are characterized as serving retention could be therapeutic (e.g., forum interactions), but it does not appear that the team intends to explicitly test this, and it is not clear what impact this might have on subsequent data analyses focused on characterizing factors that contribute to viral non-suppression.
- There are few specific plans about what the quarterly surveys and “app-based check-ins” will assess and why. Without these details, it is difficult to gauge whether the proposed work will yield findings capable of having a meaningful impact on the epidemic.

- Analyses that are the primary focus of Aim 2 appear to mostly be focused on trajectories of progression through the HIV care continuum. While potentially beneficial, few details are provided about any analyses exploring the influence of other factors on this process.
- Aim 3 focuses on developing a risk score to identify those at high-risk for viral suppression and then using that to deliver smartphone-based interventions, but very little information is provided about exactly what content these interventions would provide, how users would be encouraged to view/interact with it, and so on.
- The timeframes in terms of when data will be used to classify risk levels in Aim 3 and when interventions will begin are not clear. At least one of the primary outcomes (self-reported ART use) also has known validity issues, and the other suggests that risk levels may only be able to be characterized once every 6-months. The justification for such a low accuracy threshold for classifying those at high risk for viral suppression (AUC>0.8) is also not clear and could lead to many false alarms, risking disengagement.
- It is not clear why the outcomes of Aim 3 would include things like the System Usability Scale, given that an extensive amount of research has been focused on this system in the past, so presumably usability has already been established.

5. Environment:

Strengths

- The sponsoring environments have all of the resources necessary to support the work planned.

Weaknesses

- None were provided by the reviewer.

Study Timeline:
NOT APPLICABLE
Comments or Concerns: NOT APPLICABLE

CRITIQUE 4

Significance:	1
Investigator(s):	1
Innovation:	4
Approach:	5
Environment:	1

Overall Impact: The proposed prospective cohort study would digitally enroll a geographically diverse population of people who are likely to experience unsuppressed HIV, thereby addressing an important topic and multiple high-priority populations. The application proposes to use evidence-based and sophisticated methods for digital recruitment, retention, and interventions, including venue-based recruitment from a large network of HIV care sites, advanced digital advertising methods, and an existing app platform that the team has developed and used successfully in prior cohort studies. The team is interdisciplinary, has substantial experience establishing digital cohorts using the proposed methods, and has access to scientific environments with extensive research-related resources. Analyses appropriately account for transitions in and out of viral suppression over time. Transition milestones would ensure

adequate recruitment overall and within key subgroups, including people who are Black, rural, young, or using substances. Weaknesses include lack of innovation in the proposed methods, most of which have been applied in the investigators' prior cohort studies; lack of a transition milestone for enrollment of participants with unsuppressed HIV; and lack of details on threshold selection or evaluation of fairness in the risk prediction model. These weaknesses are minor and addressable, such that the overall potential impact of the application is considered very high.

1. Significance:

Strengths

- Research on the multilevel barriers and facilitators of viral suppression in underserved populations (e.g., Black, living in rural areas) is critical for development of interventions to improve HIV care outcomes and reduce inequities in the HIV epidemic.
- Maximizing the benefits of digital cohorts requires the use of evidence-based and sophisticated methods for promoting initial engagement and long-term retention.
- Behavioral interventions to improve HIV care outcomes are most likely to be successful when they are tailored to individuals' needs and preferences.

Weaknesses

- None noted by reviewer.

2. Investigators:

Strengths

- The large and interdisciplinary team has substantial experience using digital and other methods to recruit, retain, and intervene on diverse populations of people living with or at risk of HIV.
- Members of the team have developed and successfully used the digital platform they are proposing to leverage in this study for engagement and intervention delivery.
- Members of the team have a history of productive collaboration.

Weaknesses

- None noted by reviewer.

3. Innovation:

Strengths

- The proposed recruitment methods include advanced approaches deployed by digital advertising companies.
- Although the proposed digital methods to promote retention have been used in other populations, this study would demonstrate their utility in a cohort of people LWH.
- Use of risk prediction to tailor digital interventions by participant and time may be innovative.

Weaknesses

- Many of the proposed recruitment, retention, and intervention methods have been previously used in prior cohorts led by this team, making them evidence-based and likely to be successful but not particularly innovative.

4. Approach:

Strengths

- Leverages an existing, sophisticated, and evidence-based mobile app platform that has been used successfully by the investigative team for enrollment, engagement, data collection, and delivery of participant incentives in prior digital cohort studies.
- Multiple recruitment approaches are planned, including recruitment from a large network of HIV care sites and advanced approaches to digital advertising, to facilitate enrollment of a study population that is diverse with respect to geography and other key characteristics.
- If there are challenges recruiting 1,000 participants, the team could potentially expand recruitment to additional HIV care sites in the collaborating network.
- Analyses account for the dynamic nature of viral suppression over time.
- Transition milestones include enrollment benchmarks for people in rural areas, younger people, and people who use substances, ensuring diversity in the study population.
- Leverages existing community advisory boards throughout the proposed project.

Weaknesses

- No transition milestone is proposed for enrollment of participants with unsuppressed HIV.
- Details are not provided on threshold selection for the risk prediction model, which would determine the proportion of participants who receive the various interventions proposed.
- There is no plan to evaluate fairness in risk prediction, which could impact equity in intervention deployment.

5. Environment:

Strengths

- The scientific environment has extensive research-related resources to support the proposed study, including existing cohorts from which the team can recruit.
- The large team has been appropriately organized into subgroups based on expertise and institutional resources.
- The involvement of AIDS Healthcare Foundation, the largest HIV care network in the US, as a collaborating community partner for venue-based recruitment is a strength.

Weaknesses

- None noted by reviewer.

Study Timeline:
UNACCEPTABLE
Comments or Concerns: The timeline refers to a different study.

THE FOLLOWING RESUME SECTIONS WERE PREPARED BY THE SCIENTIFIC REVIEW OFFICER TO SUMMARIZE THE OUTCOME OF DISCUSSIONS OF THE REVIEW COMMITTEE ON THE FOLLOWING ISSUES:

PROTECTION OF HUMAN SUBJECTS: ACCEPTABLE (CODE 30)
Award can be processed, human subjects involved, no scientific review group (SRG) human subjects concerns

INCLUSION OF WOMEN PLAN: ACCEPTABLE (CODE G1A)
The inclusion plan is appropriate for the scientific goals of the research.

INCLUSION OF MINORITIES PLAN: ACCEPTABLE (CODE M1A)
The inclusion plan is appropriate for the scientific goals of the research.

INCLUSION OF INDIVIDUALS ACROSS THE LIFESPAN: ACCEPTABLE (CODE C1A)
The inclusion plan is appropriate for the scientific goals of the research.

VERTEBRATE ANIMAL: NOT APPLICABLE (CODE 10)

BIOHAZARD COMMENT: NOT APPLICABLE

SELECT AGENT: NOT APPLICABLE

RESOURCE SHARING PLANS

DATA SHARING PLAN: ACCEPTABLE
MODEL ORGANISMS SHARING PLAN: NOT APPLICABLE
GENOMIC DATA SHARING PLAN: NOT APPLICABLE

FOREIGN INSTITUTION: NOT APPLICABLE

AUTHENTICATION OF KEY BIOLOGICAL AND/OR CHEMICAL RESOURCES: NOT APPLICABLE

SCIENTIFIC REVIEW OFFICER'S ADMINISTRATIVE NOTES

CIRCUMVENTION OF PAGE LIMITS: During the review of this application, reviewers and/or NIH staff noted that this application did not comply with the page limits in the application guides and/or FOA. As per NOT-OD-11-080, NIH has the authority to withdraw applications in which certain sections are used to circumvent page limits or include inappropriate material from review or consideration for funding.

NON-COMPLIANT HYPERTEXT: This application contains non-compliant hyperlinks. Hyperlinks and URLs are only allowed when specifically noted in the funding opportunity announcement and form field instructions, as per NOT-OD-20-174. Please note that applications that do not follow these instructions may be withdrawn from review and funding consideration.

BUDGETARY OVERLAP: NOT APPLICABLE

COMMITTEE BUDGET RECOMMENDATIONS:
The budget is recommended as requested in all years.

Footnotes for 1 UG3 AI176592-01; PI Name: Gorbach, Pamina Mae

NIH has modified its policy regarding the receipt of resubmissions (amended applications).See Guide Notice NOT-OD-18-197 at https://grants.nih.gov/grants/guide/notice-files/NOT-OD-18-197.html. The impact/priority score is calculated after discussion of an application by averaging the overall scores (1-9) given by all voting reviewers on the committee and multiplying by 10. The criterion scores are submitted prior to the meeting by the individual reviewers assigned to an application, and are not discussed specifically at the review meeting or calculated into the overall impact score. Some applications also receive a percentile ranking. For details on the review process, see http://grants.nih.gov/grants/peer_review_process.htm#scoring.
